# BCL-B Promotes Lung Cancer Invasiveness by Direct Inhibition of BOK

**DOI:** 10.3390/cells14040246

**Published:** 2025-02-09

**Authors:** Palaniappan Ramesh, Amal R. Al Kadi, Gaurav M. Borse, Maximilian Webendörfer, Gregor Zaun, Martin Metzenmacher, Fabian Doerr, Servet Bölükbas, Balazs Hegedüs, Smiths S. Lueong, Joelle Magne, Beiyun Liu, Greisly Nunez, Martin Schuler, Douglas R. Green, Halime Kalkavan

**Affiliations:** 1 Department of Medical Oncology, West German Cancer Center, University Hospital Essen, University Duisburg-Essen, 45122 Essen, Germany; palaniappan.ramesh@uk-essen.de (P.R.); amalrabih.alkadi@uk-essen.de (A.R.A.K.); gauravmanik.borse@uk-essen.de (G.M.B.); maximilian.webendoerfer@uk-essen.de (M.W.); gregor.zaun@uk-essen.de (G.Z.); martin.metzenmacher@uk-essen.de (M.M.); martin.schuler@uk-essen.de (M.S.); 2 Medical Faculty, University Duisburg-Essen, 45122 Essen, Germany; fabian.doerr@rlk.uk-essen.de (F.D.); servet.boeluekbas@rlk.uk-essen.de (S.B.); balazs.hegedues@rlk.uk-essen.de (B.H.); 3 Department of Thoracic Surgery, West German Lung Center, University Medicine Essen-Ruhrlandklinik, University Duisburg-Essen, 45239 Essen, Germany; 4 German Cancer Consortium (DKTK), Partner Site University Hospital Essen, 45147 Essen, Germany; smiths-sengkwawoh.lueong@uk-essen.de; 5 Department of Immunology, St. Jude Children’s Research Hospital, Memphis, TN 38105, USA; joelle.magne@stjude.org (J.M.); beiyun.liu@stjude.org (B.L.); greisly.nunez@stjude.org (G.N.); douglas.green@stjude.org (D.R.G.); 6 BIGR, Université Paris Cité and Université des Antilles, INSERM, 75015 Paris, France; 7 National Center for Tumor Diseases (NCT) West, Campus Essen, 45122 Essen, Germany

**Keywords:** mitochondrial permeabilization, BCL-2 family, BCL-B, BOK, DTP, persister phenotype, EMT, invasiveness, drug-resistance, cancer

## Abstract

Expression of *BCL-B*, an anti-apoptotic BCL-2 family member, is correlated with worse survival in lung adenocarcinomas. Here, we show that BCL-B can mitigate cell death initiation through interaction with the effector protein BOK. We found that this interaction can promote sublethal mitochondrial outer membrane permeabilization (MOMP) and consequently generate apoptosis-flatliners, which represent a source of drug-tolerant persister cells (DTPs). The engagement of endothelial-mesenchymal-transition (EMT) further promotes cancer cell invasiveness in such DTPs. Our results reveal that BCL-B fosters cancer cell aggressiveness by counteracting complete MOMP.

## 1. Introduction

The binding partners and functions of most BCL-2 family members have been studied rigorously in the past decades. Yet, some BCL-2 family proteins remain enigmatic concerning their function and impact in cancer.

In general, the B-cell lymphoma-2 (BCL-2) family consists of various proteins with either anti- or pro-apoptotic properties. The BCL-2 family effector proteins BAX, BAK, and BOK can form pores which lead to mitochondrial outer membrane permeabilization (MOMP) and thereby initiate the intrinsic pathway of apoptosis [1]. Anti-apoptotic BCL-2 family members can bind and inhibit effector proteins. Under stress conditions, pro-apoptotic BH3-only proteins interact via their BH3 domains either with anti-apoptotic proteins or directly with BAX and BAK to promote MOMP [2,3]. Thus, BH3 mimetics, such as Navitoclax, Venetoclax, and MCL-1 inhibitors have been exploited for cancer therapy and have proven clinical activity in some leukemias and lymphomas [4,5].

BCL-B (also known as BCL2-L10 and NRH, or Boo and Diva in mice), was discovered as an anti-apoptotic BCL-2 family protein that harbors all four BCL-2 homology (BH) domains (BH1, BH2, BH3, and BH4) as well as the terminal transmembrane domain [6]. Most anti-apoptotic BCL-2 family proteins have been described as tumor promoters or even oncogenes. However, contradicting data characterize BCL-B either as an oncogene or as a tumor-suppressor [7]. Yet, BCL-B is highly expressed in many cancers, and has been uncovered as a predictive factor for therapy resistance in hematological cancers [8,9,10,11]. Surprisingly, in vitro BCL-B was only a weak inhibitor of cell death as compared to other prosurvival BCL-2 members, indicating a distinct role for this protein [6,10].

The effector protein Bcl-2-related ovarian killer (BOK) is another conundrum of the BCL-2 family [12]. Being frequently deleted in cancers, BOK has been identified as a tumor-suppressor [13]. However, its functional role in inducing MOMP was mostly described in the context of ER-associated degradation (ERAD), where cell lines expressing “unstable” BOK sustain survival by active degradation of this MOMP effector [14]. Consequently, often viable cell lines (MEF, HCT116) show low or barely detectable protein levels of BOK [15], unless ER-associated degradation (ERAD) is disrupted, e.g., by proteasome inhibition.

Survivors of apoptotic stimuli are drug-tolerant persister cells (DTPs) that have characteristics distinct from those of the untreated, parental cells (control). These characteristics of the DTPs change over time to again resemble the parental cells upon withdrawal of the apoptotic stimulus. We previously found that engagement of the apoptotic process, i.e., release of cytochrome c upon sublethal MOMP, is necessary and sufficient for the characteristics of the DTPs [16]. We have termed such cells flatliners to indicate that they had engaged the cell-death machinery but survived. We suggest that flatliners are DTPs, but not all DTPs are necessarily flatliners. Importantly, the occurrence of MOMP followed by cell survival can direct a phenotypic switch towards most aggressive characteristics of cancer cells, such as metastatic capacity, cross-resistances towards therapies, and changes in proliferative or secretory behavior [16,17,18]. Consequently, DTPs are of particular interest for our understanding of minimal residual disease (MRD) and cancer relapse with metastatic outbursts.

Here, we reveal that BCL-B can bind BOK and counteract its contribution to MOMP and cell death, which consequently leads to DTP generation. Cancer cells that stably express BOK rely to a significant extent on this effector protein for the execution of apoptosis in response to select cytotoxic drugs. Further, we found that the presence of BCL-B and its inhibition of BOK, can promote sublethal MOMP and thereby foster cancer cell invasiveness, relayed by EMT.

## 2. Materials and Methods

### 2.1. Cells

The human lung cancer cell line PC9 was purchased from Millipore Sigma (US and Canada, Cat# 90071810). A549, another human lung cancer cell line, was kindly provided by Richard J. Webby (St. Jude Children’s Research Hospital). Human uveal melanoma cell lines 92.1 and UPMD1 were a gift from Klaus Griewank (University Hospital Essen). Human embryonic kidney cells (HEK) and human cancer cell lines HCT116 (colorectal) and H226 (lung squamous cell carcinoma) were purchased from ATCC. *Bax* and *Bak* DKO murine embryonic fibroblasts (MEF) expressing doxycycline inducible *Bok*, have been previously described [18].

Cells were maintained in complete RPMI media (5% fetal bovine serum (FBS), 2 mM L-glutamine, and 100 units/mL penicillin-streptomycin). MEF culture medium was supplemented with nonessential amino acids, 1 mM sodium pyruvate, and 55 μM β-mercaptoethanol. All cells used in this study were cultivated at 37 °C with 5% CO_2_. The generation of knock-out cells was performed with CRISPR-Cas9 technology as previously described [14]. Using the MycoAlert Mycoplasma Detection kit (Lonza, Visp, Switzerland, #LT07), we confirmed that the cell lines used in these studies are mycoplasma negative.

### 2.2. Transient Transfection

Transient transfection with DNA and siRNA were performed for 48 h using ViafectTM (Promega, Madison, WI, USA) for HEK and A549, Fugene^R^HD (Promega) for PC9, A549, and MEF, and Lipofectamine™ RNAiMAX transfection reagents, respectively. Cells were transfected when 50–75% confluent using serum-free Opti-MEM (Invitrogen, Waltham, MA, USA) as per the manufacturers’ instructions. The siRNA oligos had been purchased from horizon as ON-TARGETplus siRNA pools of 4 oligos (ON-TARGETplus Non-targeting Control Pool, Catalog ID:D-001810-10-20 and ON-TARGETplus Human BCL2L10 siRNA, Catalog ID: L-004382-00-0005).

### 2.3. Flow Cytometry

For intracellular staining, cells were first fixed and then permeabilized according to the manufacturer’s instructions using the Intracellular Fixation & Permeabilization Buffer Set (BD). To quantify cells that had undergone MOMP and cytochrome c release into the cytosol, cells were incubated with 20 µg/mL digitonin in PBS (Sigma-Aldrich, St. Louis, MO, USA) before fixation and further staining procedure. Cytochrome c Alexa Fluor^®^ 647 from BioLegend (San Diego, CA, USA) Cat# 612310 was used in a 1:200 dilution.

For cell death quantification with flow cytometry, cells were treated with indicated drugs, harvested, and analyzed after staining with ViaDye™ Red Fixable Viability Dye (CYTEK Biosciences, Fremont, CA, USA, Cat# R7-60008) as per the manufacturers’ instructions. The following drugs have been used in experiments: Tunicamycin (Sigma, Livonia, MI, USA, Cat# T7765, 10 µM), Paclitaxel (MedChemExpress, Monmouth Junction, NJ, USA, Cat# HY-B0015, 5 µM), Vinblastin (Sigma, USA, Cat# V1377, 5 µM), MG132 (Sigma, USA, Cat# C2211, 10 µM), Bortezomib (MedChemExpress, USA, Cat# HY-10227, 1 µM), and Cisplatin (MedChemExpress, USA, Cat# HY-17394, 10 µM).

Data acquirement and analysis was performed in a spectral analyzer (CYTEK Aurora 5-laser) and data were processed using FlowJo version 10 software (Tree Star).

### 2.4. IncuCyte Analysis

Cell-death kinetics were detected by the IncuCyte S3 or SX-5 imaging system (Essen Bioscience/Sartorius, Ann Arbor, MI, USA). Dead cells were stained with 400 ng/mL propidium iodide (PI) or 25 nM SYTOX Green (Invitrogen). Quantification of SYTOX Green- or PI-positive cells was performed by the IncuCyte image analysis software (version 2022B, Essen Bioscience/Sartorius). Data were expressed as the fold change in positive events per well. Error bars represent the standard deviation (SD) from the mean for replicate samples.

For transwell assays, ClearView Plates were precoated with extracellular matrix of 50 μg/mL Collagen 1 and incubated for 1 h, before cells were seeded into the insert and plates were further prepared according to the manufacturer’s protocol. For MMP treatment, 20 µM GM6001 (MERCK, Darmstadt, Germany, Cat#36420) was added to cells. Note: The y-axes on the microplates graphs are normalized bottom-view cell counts and the assay duration represented is 72 h.

### 2.5. Western Blotting

Cells were lysed in complete cell lysis buffer (50 mM Tris-Cl pH 7.4, 150 mM NaCl, cOmplete^®^ protease inhibitors cocktail (Roche, Basel, Switzerland), and 0.5% NP-40) and denaturated in sample buffer (BioRad, Hercules, CA, USA) containing DTT. The protein concentration of cell lysates was measured by the BCA assay (Pierce, Appleton, WI, USA) and systematically normalized before Western blotting. A total of 20 µg of protein was loaded per sample. The following antibodies were used for Western blotting: anti-BAX CST (USA) #5023, anti-BAK CST 3814, anti-BOK abcam (UK) ab186745, BCL-B CST #3869, ß-actin Santa Cruz (USA) sc-47778-HRP, Vimentin CST #5741, and ZEB1 CST #3396. Peroxidase Conjugated ImmunoPure Goat Anti-Rabbit IgG (H+L) from Pierce/Thermo Scientific (Waltham, MA, USA, Cat# 31460) has been used as secondary antibody.

### 2.6. Immunoprecipitation

HEK cells were transiently transfected with constructs carrying human BOK and flag-tagged anti-apoptotic BCL-2 family proteins. Once 24 h had passed after transfection, cells were treated with 10 µM MG132 for 4 h, washed in ice-cold PBS, trypsinized, and then again washed in PBS. Briefly, cells were lysed in cell lysis buffer (see above). Clarified lysates were immunoprecipitated with anti-FLAG^®^-M2 affinity gel (A2220, Sigma-Aldrich) incubated for at least 2 h at 4 °C, and complexes were washed 5 times in cell lysis buffer. Immunoprecipitates were eluted from the anti-FLAG^®^-M2 affinity gel by incubating with the FLAG^®^ Peptide (100 μg/mL; F3290, Sigma-Aldrich).

### 2.7. Gene Expression and Survival Analysis

For BCL-B gene expression comparison between human normal and cancer samples we utilized RNA-sequencing expression data of the indicated numbers of tumors and normal samples from the TCGA (please see legends of the figures) and the GTEx projects, which were analyzed by the Webtool GEPIA2 [18].

Kaplan–Meier plots were generated by use of the online webtool “Kaplan-Meier Plotter” using univariate Cox regression analysis. Respective patients’ cohorts were split by auto-select best cutoff for BCL-B expression using datasets for gastric cancer [19,20,21], breast cancer [22,23,24], colorectal cancer [25], pancreatic ductal cancer [26], and non-small-cell lung cancer [27].

### 2.8. Statistical Analysis

Please refer to the figure legends for a more detailed description of sample sizes and statistical tests that have been performed. For in vitro studies, data are plotted as means ± standard deviations (SD). Each figure shows the results of one experiment on which the statistics are based. When 2-way-ANOVA was applied to determine significance, this was followed by Šidák’s multiple comparisons test. F-statistics from interaction factors are shown within respective graphs. Post hoc F-test analysis using g*power revealed a statistical power with values in the range of 0.82 to 1 for all ANOVA analyses performed in respective experiments of this manuscript [19,20].

All data were plotted and analyzed with GraphPad Prism 9.0 software and R version 4.4.1. All graphs from in vitro experiments show data from one representative experiment of the three independent experiments performed.

## 3. Results

### 3.1. BCL-B Impedes Apoptosis in a BOK-Dependent Manner

**Figure 1:**
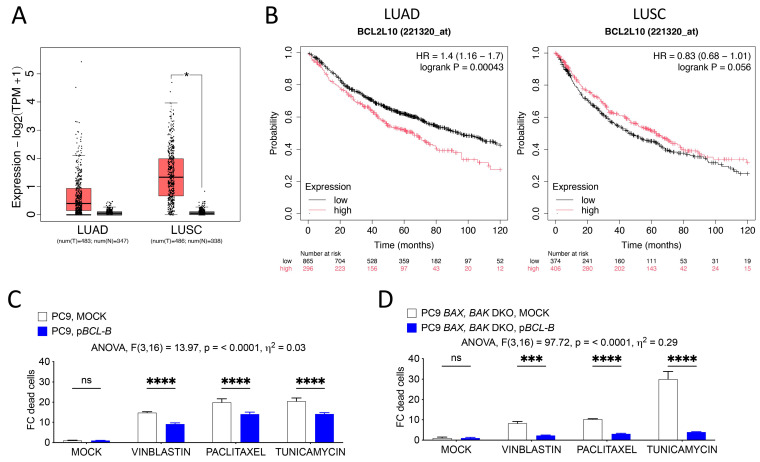
BCL-B is highly expressed in lung cancers and inhibits apoptosis in a BAX/BAK-independent manner. (**A**) BCL-B expression in lung adenocarcinoma (LUAD) and lung squamous cell cancer (LUSC) (T, red bars) compared to normal tissue (N, gray bars). * *p* < 0.05 (ANOVA). (**B**) Kaplan–Meier survival curve of patients with indicated cancers belonging to high or low BCL-B expression from the TCGA database (auto cut-off). *p*-values are indicated. (**C**) Fold change in the number of dead WT PC9 cells or (**D**) *BAX BAK* DKO PC9 cells treated with indicated drugs and with or without overexpression of BCL-B. Mean ± SD of n = 3 samples per condition are shown. Graphs represent one of three independent experiments. Two-way ANOVA with Šidák’s multiple comparisons test: *** *p* < 0.001; **** *p* < 0.0001; ns, not significant (*p* > 0.05).

*BCL-B* is highly expressed in various cancer types with high interpatient variability when compared to normal tissue (Figure 1A and Figure S1). For instance, patients with lung adenocarcinoma (LUAD) frequently show upregulated *BCL-B*, which correlates with worse survival (Figure 1B). Although patients with lung squamous cell cancer (LUSC) show significantly increased BCL-B expression, it does not correlate with overall patient survival. To gain mechanistical insights as to how the anti-apoptotic and putative tumor-promoting potential of BCL-B in cancer cells might be exerted, we first focused on the PC9 lung adenocarcinoma cell line in our following studies. We transfected PC9 cells transiently with *BCL-B* and exposed cells to diverse cytotoxic drugs (Figure 1C). Indeed, overexpression of *BCL-B* could diminish cell death to a significant level. Since drug-induced cytotoxicity is mostly but not exclusively relayed by BAX activation [16] and interaction of BAX with BCL-B had been described in overexpression experiments in MEF and HEK cells [21,22], we analyzed the impact of *BAX* and *BAK* double knock-out (DKO) on drug-induced cell death of PC9 cells. Surprisingly, not only did we observe a considerable induction of cell death but also a remarkable suppression of death by *BCL-B* overexpression in PC9 DKO cells, which exceeded the extent of protection observed in WT cells.

**Figure 2:**
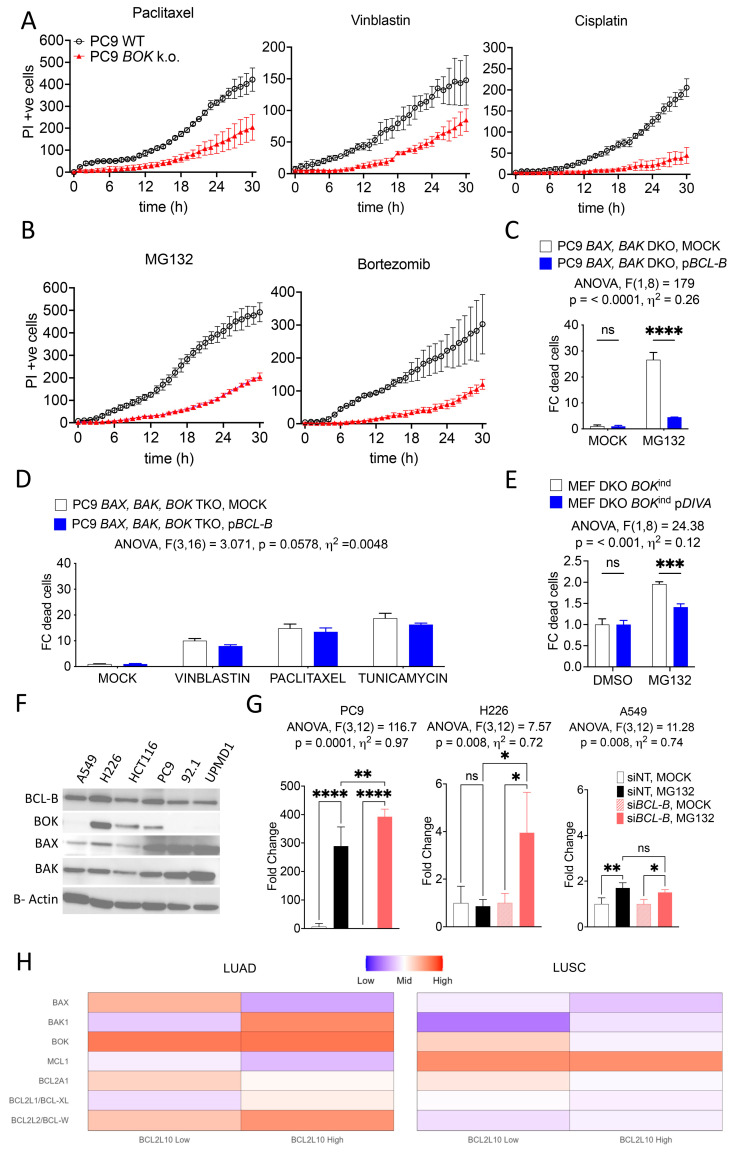
BCL-B inhibits BOK-dependent cell death. (**A**,**B**) Live cell imaging and detection of PI positive WT or *BOK* k.o. PC9 cells that have been treated with or without indicated drugs. Mean ± SD from n = 3 samples per group are shown. (**C**) Fold change in number of dead *BAX BAK* DKO PC9 cells treated with MG132 and with or without overexpression of *BCL-B*. Mean ± SD from n = 3 samples per condition are shown. Two-way ANOVA with Šidák’s multiple comparisons test: **** *p* < 0.0001; ns, not significant (*p* > 0.05). (**D**) Fold change in the number of dead *BAX BAK BOK* TKO PC9 cells treated with indicated drugs and with or without overexpression of *BCL-B*. Mean ± SD of n = 3 samples per condition are shown. Two-way ANOVA. (**E**) Fold change quantification of dead *Bax Bak* DKO MEF cells harboring doxycycline-inducible Bok, treated with indicated drugs with or without overexpression of murine *Bcl-B* (*Diva*). Mean ± SD of n = 3 samples per condition are shown. Two-way ANOVA with Šidák’s multiple comparisons test: *** *p* < 0.001; ns, not significant (*p* > 0.05). (**F**) Immunoblot with the indicated antibodies of cytosolic extracts from various indicated cell lines. (**G**) Fold change in the number of indicated cell lines silenced with either non-targeted control or *BCL-B* directed siRNAs before treatment with or without MG132. Mean ± SD of n = 3 samples per condition are shown. One-way ANOVA with Tukey’s multiple comparisons test: **** *p* < 0.0001; ** *p* < 0.01; * *p* < 0.05; ns, not significant (*p* > 0.05). (**A**–**G**) All graphs represent one of three independent experiments. (**H**) Normalized BCL2 family gene expression in lung adenocarcinoma (LUAD) and lung squamous cell carcinoma (LUSC) of the TCGA pancancer cohort. Expression was normalized within LUAD and LUSC, respectively, following RNA-transcript processing using RSEM (RNA-Seq by Expectation-Maximization).

Apart from BAX and BAK, BOK is a BCL-2 effector protein that has been widely neglected in the context of drug-induced cytotoxicity. We generated BOK-deficient PC9 cells and tested their susceptibility to death induced by various drugs in comparison to WT cells. Strikingly, BOK-ablation diminished cell death induced by Paclitaxel, Vinblastin, and Cisplatin (Figure 2A and Figure S2). Since BOK is relevant in ER-stress induced cell death [18], we also confirmed that the proteasome-inhibitors MG132 and Bortezomib were mostly inefficient in inducing cell death in our BOK-deficient cell lines (Figure 2B). Interestingly, we were able to almost completely suppress MG132-induced cell death by overexpression of *BCL-B* in *BAX* and *BAK* DKO PC9 cells (Figure 2C), further indicating that BCL-B exerts its anti-apoptotic function via inhibition of BOK. To extend this observation under different therapeutic conditions, we tested the impact of *BCL-B* overexpression in *BAX*, *BAK*, and *BOK* triple knock-out (TKO) PC9 cells. In line with our findings so far, *BCL-B* overexpression poorly suppressed cell death in the absence of BCL-2 family effector proteins (Figure 2D). To further test the idea that BCL-B inhibits BOK-dependent cell death, we made use of *Bax*, *Bak* DKO murine embryonic fibroblasts (MEF) which express a doxycycline-inducible *Bok*. Since MEF do not express *Bok* endogenously, cell death is maximized when cells are treated with doxycycline before MG132 application [18]. Overexpression of *DIVA* (murine *Bcl2l10*) was able to significantly inhibit BOK-induced cell death (Figure 2E). Knowing that *BOK* is frequently deleted in cancers, while *BCL-B* is often amplified, we next aimed to identify cell lines with detectable endogenous levels of both proteins. Studying various cancer cell lines, we identified by Western blotting that the lung cancer cell lines A549 (adenocarcinoma), H226 (squamous cell carcinoma), and PC9 (adenocarcinoma) showed high variability in BOK protein levels, while all of them had comparably high BCL-B (Figure 2F). Next, we induced apoptosis in all these lung cancer cell lines, with or without previous silencing of *BCL-B* (Figure 2G). PC9 cells were most responsive towards MG132 treatment. In these cells, BOK expression is moderate, and BCL-B could make a partial but significant difference in cell death. Cell line H226, which had revealed the highest expression of BOK and BCL-B, showed complete protection against MG132-induced cell death in the presence of BCL-B. Finally, A549, which lacks endogenous BOK, showed overall low sensitivity towards MG132 which was independent of the presence of BCL-B. Taken together, our data reveal that BCL-B is an anti-apoptotic BCL-2 family member which inhibits the pro-apoptotic function of the effector protein BOK.

Next, we checked expression levels of the effectors *BOK*, *BAX*, and anti-apoptotic BCL-2 family members in LUAD, LUSC, and breast cancer (BRCA). Interestingly, expression of both *BOK* and *BAX* was suppressed in LUSC when compared to LUAD and BRCA, while *MCL1* expression was mostly increased (Figure 2H and Figure S1). This finding poses a possible explanation for the non-evident difference in *BCL-B*-associated overall survival in LUSC patients, while the other cancer entities show a significant correlation.

### 3.2. BCL-B Directly Interacts with BOK

**Figure 3:**
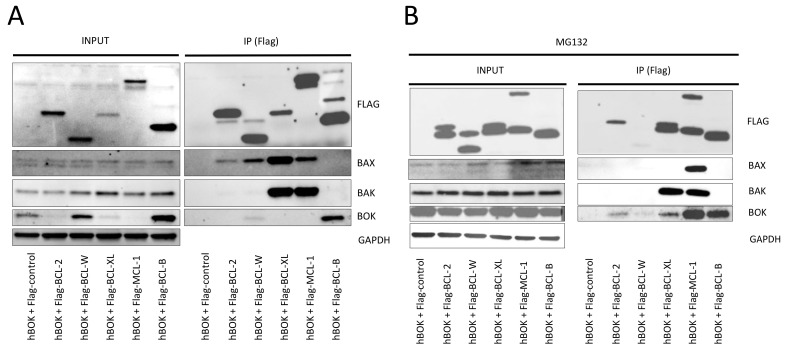
BCL-B binds to BOK. Co-immunoprecipitation experiments in cell lysates generated from HEK cells transfected with indicated flag-tagged anti-apoptotic human BCL-2 family members together with human BOK (hBOK) without (**A**) or with (**B**) treatment with MG132.

To elucidate if BCL-B mediated inhibition of BOK might be conferred via direct interaction between the two proteins, we performed co-immunoprecipitation experiments in HEK cells expressing flag-tagged anti-apoptotic proteins combined with overexpression of *BOK*. We observed co-immunoprecipitation of BOK with flag-tagged BCL-B (Figure 3A). However, a previously described interaction between BOK and MCL-1 was hardly detected under these conditions [23,24,25]. Proteasomal degradation is a crucial mediator of protein stability for BOK as well as MCL-1 and BCL-B and might affect protein–protein interactions and function [8,10,26]. Consequently, we repeated the same co-immunoprecipitation experiments after pretreatment with the proteasome inhibitor MG132. BOK co-immunoprecipitation was most evident in combination with BCL-B and MCL-1 (Figure 3B).

### 3.3. BCL-B Promotes Sublethal MOMP in DTPs

**Figure 4:**
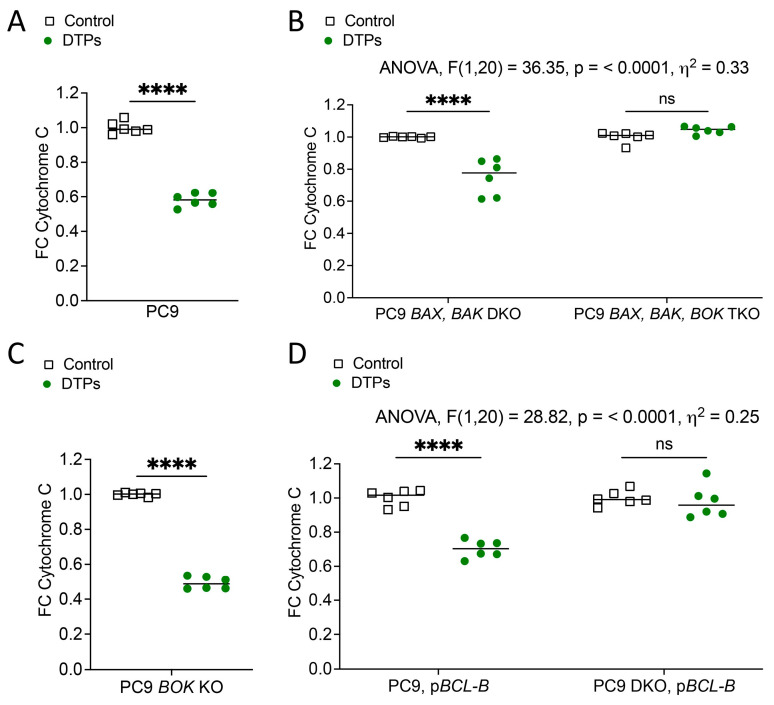
BCL-B promotes sublethal MOMP in DTPs. Fold change quantification of cytochrome c positive cells in control and DTP cells generated with MG132 (**A**) in WT PC9 or (**B**) *BAX BAK* DKO versus *BAX BAK BOK* TKO PC9 or (**C**) *BOK* k.o. PC9 cells or (**D**) indicated cell lines with overexpression of *BCL-B*. n = 6 samples are shown per condition. Unpaired *t*-test (**A**,**C**) or two-way ANOVA (**B**,**D**) with Šidák’s multiple comparisons test: **** *p* < 0.0001; ns, not significant (*p* > 0.05). All graphs represent one of three independent experiments.

Our data so far reveals that BCL-B can promote cancer cell survival during cytotoxic treatment. However, in the presence of all BCL-2 effector proteins, BAX, BAK, and BOK, the overall anti-apoptotic impact of BCL-B is quite limited (Figure 1C,D). Thus, we wondered if inhibition or deletion of only one or two of the three effector proteins might promote partial MOMP with sublethal apoptosis engagement, thereby promoting cancer cell survival and aggressiveness [27]. To be able to retrospectively identify cells that had survived treatment despite MOMP engagement, we determined the reduction in cytochrome c on a cellular level as a surrogative marker that MOMP had occurred. As expected, DTPs generated from WT PC9 cells treated with MG132 indeed showed diminished cytochrome c levels, indicating that MOMP has taken place in DTPs (Figure 4A). Next, we tested the occurrence of MOMP in PC9 *BAX, BAK* DKO and *BAX, BAK, BOK* TKO cells. Again, a reduction in cytochrome c was found in PC9 DKO DTPs, even though the extent of cytochrome c loss was less than in WT, since these cells only harbor BOK but not the other two BCL-2 effector proteins. However, cytochrome c protein levels were maintained in TKO cells that were treated with MG132, since MOMP cannot proceed in the absence of all three effector proteins (Figure 4B). In the absence of BOK alone, cytochrome c was diminished in DTPs (Figure 4C), indicating that MOMP can take place in response to MG132 even in the absence of BOK, even though cell death in response to proteasome inhibition is strongly dependent on BOK (Figure 2B,C). Yet, we know that dependencies might change in the absence or presence of BCL-2 family proteins (Figure 1C,D). Strikingly, the overexpression of BCL-B, which promoted cell survival upon treatment (Figure 1C), led to DTPs that had engaged MOMP in WT PC9 cells but not in PC9 *BAX, BAK* DKO cells (Figure 2D). This finding further confirms that BCL-B inhibits BOK-dependent MOMP execution and indicates that the loss or inhibition of either BCL-2-family effector protein (but not all) can promote sublethal MOMP and the generation of DTPs.

### 3.4. Cancer Cell Invasiveness Is Promoted in DTPs

**Figure 5:**
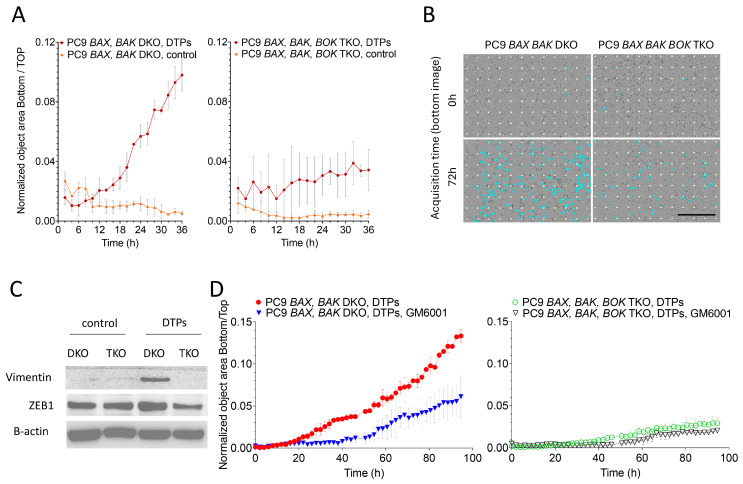
EMT and cancer cell invasiveness is promoted in DTPs. (**A**) Live cell imaging analysis and (**B**) bottom transwell images of transmigrated *BAX BAK* DKO and *BAX BAK BOK* TKO PC9 control or DTP cells generated with MG132. Mean ± SD from n = 2 samples per group are shown. Counted cells are marked by blue mask. Scale bar 700µm. (**C**) Immunoblots of cell lysates from *BAX BAK* DKO and *BAX BAK BOK* TKO PC9 control or DTP cells treated with MG132 using indicated antibodies. (**D**) Incucyte analysis of transmigrated *BAX BAK* DKO and *BAX BAK BOK* TKO PC9 control or DTP cells generated with MG132, and treated with or without GM6001 20 µM. Mean ± SD from n = 2 samples per group are shown. All graphs represent one of three independent experiments.

Substantial evidence exists concerning engagement of EMT in cancer cells that have engaged sublethal apoptosis [16,27,28,29]. Invasive tumor growth is a characteristic of aggressive cancers. The overall enhanced metastatic capacity of DTPs suggests that cancer cells that undergo sublethal MOMP exert an invasive phenotype. To test this hypothesis, we performed a transwell-assay comparing control and DTPs generated only in the presence of BOK, but not BAX and BAK, with a strong barrier composed of Collagen 1. Strikingly, only *BAX, BAK* DKO-DTPs, but not TKO-PS, were able to invade and pass through the collagen barrier and migrate to the bottom layer of the transwell (Figure 5A,B). Assuming that the activation of EMT promoted invasiveness, we tested for the expression of EMT transcription factors and the mesenchymal markers. Interestingly, ZEB1 was upregulated in DKO-DTPs which was associated with the expression of Vimentin (Figure 5C). Since the upregulation of matrix-metalloproteases was evident in DTPs (Figure S3), we thought that matrix-metalloproteases (MMP) might enable cell invasiveness by degradation of Collagen 1. Thus, we tested the impact of an MMP-inhibitor (GM6001) on DTP invasiveness in our transwell-assay. Indeed, transmembrane cancer cell migration was diminished upon MMP-inhibition (Figure 5D).

## 4. Discussion

Herein we found that BCL-B is an anti-apoptotic protein which can interact with and inhibit the BCL-2 effector protein BOK. This finding is of interest in scenarios where *BOK* is expressed and critically involved in engagement of the apoptotic machinery. We reveal several cytotoxic drugs and proteasome inhibitors which at least in part confer cell death via the activation of BOK and can be inhibited by BCL-B. Importantly, we provide evidence that sublethal MOMP can occur, even if only one of the three BCL-2 effector proteins, BAX, BAK, or BOK, is inhibited. As a major consequence of sublethal MOMP, EMT is promoted during the generation of the DTPs, which fosters an invasive phenotype of cancer cells. This finding is in line with previous studies where an EMT signature was described in DTPs [30,31]. Both EMT as well as sublethal MOMP have been identified as drivers of an increased metastatic capacity of cancer cells [27,28,32,33,34]. Our data add mechanistical evidence as to how imbalances between BCL-2 family members can contribute to cancer cell invasiveness as part of the metastatic process [34]. Moreover, our findings further underline the notion that multidomain BCL-2 effector proteins are not entirely redundant [35,36]. In line with our discoveries, independent work has shown that experimental overexpression of *BOK* in A549 lung adenocarcinoma cell line inhibits migration and endothelial-mesenchymal transition (EMT) [37].

Previous work provided evidence from overexpression experiments in MEF or HEK, with tagged *BAX* and *BAK*, for binding selectivity of BCL-B towards BAX and not BAK [6,21,22]. However, this was not evident in our co-immunoprecipitation experiments in the presence of BOK. We suspect that binding affinity is context-dependent, as has been shown for diverse other BCL-2 family members [38], and may be determined by the expression or absence of certain BCL-2 family proteins. For instance, *BOK* is frequently deleted in cancers while *MCL-1* is often amplified [13]. This observation may add to our understanding as to why survival analysis does not necessarily correlate with *BCL-B* expression in some models, despite its function as an anti-apoptotic protein and its high expression levels in some cancers. In our studies, we found that *BOK* expression is often downregulated in LUSC, where *BCL-B* expression does not correlate with survival, while *BOK* is expressed in LUAD, where *BCL-B* expression levels significantly correlate with overall survival. Our results provide a plausible explanation for this finding, since apoptosis might be relayed more dominantly via BOK in LUAD, which further accentuates a tumor promoting role of BCL-B in this specific cancer biology.

Our aim in this study was to investigate the role of BCL-B in cancer cells. To limit multifactorial perturbations and strictly dissect the function of BCL-B in the regulation of apoptosis, we performed well controlled in vitro studies. While we show that BCL-B’s major interaction partner BOK plays a role in diverse drug treatments, we have focused mainly on MG132 for the generation of DTPs due to its strong BOK-dependent engagement of apoptosis. It will be crucial to determine how our findings apply to DTPs generated with other drugs, in vivo models, or patients with residual disease in future investigations.

## Data Availability

The original contributions presented in this study are included in the article/supplementary material. Further inquiries can be directed to the corresponding author.

## Supplementary Materials

The following supporting information can be downloaded at: https://www.mdpi.com/article/10.3390/cells14040246/s1, Figure S1: BCL-B is highly expressed in cancers and correlates with survival in select tumor types. Figure S2: Generation of BOK k.o. cells. Figure S3: DTPs express EMT markers.

## References

[1] Bhola P., Letai A. (2016). Mitochondria—Judges and Executioners of Cell Death Sentences. Mol. Cell.

[2] Garrido C., Galluzzi L., Brunet M., Puig P.E., Didelot C., Kroemer G. (2006). Mechanisms of Cytochrome c Release from Mitochondria. Cell Death Differ..

[3] Tait S.W.G., Green D.R. (2013). Mitochondrial Regulation of Cell Death. CSH Perspect. Biol..

[4] Merino D., Kelly G.L., Lessene G., Wei A.H., Roberts A.W., Strasser A. (2018). BH3-Mimetic Drugs: Blazing the Trail for New Cancer Medicines. Cancer Cell.

[5] Kalkavan H., Green D.R. (2018). MOMP, Cell Suicide as a BCL-2 Family Business. Cell Death Differ..

[6] Ke N., Godzik A., Reed J.C. (2001). Bcl-B, a Novel Bcl-2 Family Member That Differentially Binds and Regulates Bax and Bak. J. Biol. Chem..

[7] Pervushin N.V., Kopeina G.S., Zhivotovsky B. (2023). Bcl-B: An “Unknown” Protein of the Bcl-2 Family. Biol. Direct.

[8] van de Kooij B., Rooswinkel R.W., Kok F., Herrebout M., de Vries E., Paauwe M., Janssen G.M.C., van Veelen P.A., Borst J. (2013). Polyubiquitination and Proteasomal Turnover Controls the Anti-Apoptotic Activity of Bcl-B. Oncogene.

[9] Duong M.Q., Gadet R., Treilleux I., Borel S., Nougarède A., Marcillat O., Gonzalo P., Mikaelian I., Popgeorgiev N., Rimokh R. (2023). Nrh L11R Single Nucleotide Polymorphism, a New Prediction Biomarker in Breast Cancer, Impacts Endoplasmic Reticulum-Dependent Ca^2+^ Traffic and Response to Neoadjuvant Chemotherapy. Cell Death Dis..

[10] Rooswinkel R.W., van de Kooij B., de Vries E., Paauwe M., Braster R., Verheij M., Borst J. (2014). Antiapoptotic Potency of Bcl-2 Proteins Primarily Relies on Their Stability, Not Binding Selectivity. Blood.

[11] Cluzeau T., Robert G., Mounier N., Karsenti J.M., Dufies M., Puissant A., Jacquel A., Renneville A., Preudhomme C., Cassuto J.-P. (2012). BCL2L10 Is a Predictive Factor for Resistance to Azacitidine in MDS and AML Patients. Oncotarget.

[12] Brem E.A., Letai A. (2016). BOK: Oddball of the BCL-2 Family. Trends Cell Biol..

[13] Beroukhim R., Mermel C.H., Porter D., Wei G., Raychaudhuri S., Donovan J., Barretina J., Boehm J.S., Dobson J., Urashima M. (2010). The Landscape of Somatic Copy-Number Alteration across Human Cancers. Nature.

[14] Llambi F., Wang Y.-M., Victor B., Yang M., Schneider D.M., Gingras S., Parsons M.J., Zheng J.H., Brown S.A., Pelletier S. (2016). BOK Is a Non-Canonical BCL-2 Family Effector of Apoptosis Regulated by ER-Associated Degradation. Cell.

[15] Echeverry N., Bachmann D., Ke F., Strasser A., Simon H.U., Kaufmann T. (2013). Intracellular Localization of the BCL-2 Family Member BOK and Functional Implications. Cell Death Differ..

[16] Kalkavan H., Chen M.J., Crawford J.C., Quarato G., Fitzgerald P., Tait S.W.G., Goding C.R., Green D.R. (2022). Sublethal Cytochrome c Release Generates Drug-Tolerant Persister Cells. Cell.

[17] Ichim G., Tait S.W.G. (2016). A Fate Worse than Death: Apoptosis as an Oncogenic Process. Nat. Rev. Cancer.

[18] Victorelli S., Salmonowicz H., Chapman J., Martini H., Vizioli M.G., Riley J.S., Cloix C., Hall-Younger E., Espindola-Netto J.M., Jurk D. (2023). Apoptotic Stress Causes MtDNA Release during Senescence and Drives the SASP. Nature.

[19] Faul F., Erdfelder E., Lang A.-G., Buchner A. (2007). G*Power 3: A Flexible Statistical Power Analysis Program for the Social, Behavioral, and Biomedical Sciences. Behav. Res. Methods.

[20] Faul F., Erdfelder E., Buchner A., Lang A.-G. (2009). Statistical Power Analyses Using G*Power 3.1: Tests for Correlation and Regression Analyses. Behav. Res. Methods.

[21] Zhai D., Jin C., Huang Z., Satterthwait A.C., Reed J.C. (2008). Differential Regulation of Bax and Bak by Anti-Apoptotic Bcl-2 Family Proteins Bcl-B and Mcl-1. J. Biol. Chem..

[22] Rautureau G., Yabal M., Yang H., Huang D., Kvansakul M., Hinds M. (2012). The Restricted Binding Repertoire of Bcl-B Leaves Bim as the Universal BH3-Only Prosurvival Bcl-2 Protein Antagonist. Cell Death Dis..

[23] Kalkat M., Garcia J., Ebrahimi J., Melland-Smith M., Todros T., Post M., Caniggia I. (2013). Placental Autophagy Regulation by the BOK-MCL1 Rheostat. Autophagy.

[24] Szczesniak L.M., Bonzerato C.G., Wojcikiewicz R.J.H. (2021). Identification of the Bok Interactome Using Proximity Labeling. Front. Cell Dev. Biol..

[25] Lucendo E., Sancho M., Lolicato F., Javanainen M., Kulig W., Leiva D., Duart G., Andreu-Fernández V., Mingarro I., Orzáez M. (2020). Mcl-1 and Bok Transmembrane Domains: Unexpected Players in the Modulation of Apoptosis. Proc. Natl. Acad. Sci. USA.

[26] Schulman J.J., Wright F.A., Kaufmann T., Wojcikiewicz R.J. (2013). The Bcl-2 Protein Family Member Bok Binds to the Coupling Domain of Inositol 1,4,5-Trisphosphate Receptors and Protects Them from Proteolytic Cleavage. J. Biol. Chem..

[27] Berthenet K., Ferrer C., Fanfone D., Popgeorgiev N., Neves D., Bertolino P., Gibert B., Hernandez-Vargas H., Ichim G. (2020). Failed Apoptosis Enhances Melanoma Cancer Cell Aggressiveness. Cell Rep..

[28] Fanfone D., Wu Z., Mammi J., Berthenet K., Neves D., Weber K., Halaburkova A., Virard F., Bunel F., Jamard C. (2022). Confined Migration Promotes Cancer Metastasis through Resistance to Anoikis and Increased Invasiveness. eLife.

[29] Sun G., Guzman E., Balasanyan V., Conner C.M., Wong K., Zhou H.R., Kosik K.S., Montell D.J. (2017). A Molecular Signature for Anastasis, Recovery from the Brink of Apoptotic Cell Death. J. Cell Biol..

[30] Nieto M.A. (2011). The Ins and Outs of the Epithelial to Mesenchymal Transition in Health and Disease. Annu. Rev. Cell Dev. Biol..

[31] Shen S., Vagner S., Robert C. (2020). Persistent Cancer Cells: The Deadly Survivors. Cell.

[32] Kalkavan H., Rühl S., Shaw J.J.P., Green D.R. (2023). Non-Lethal Outcomes of Engaging Regulated Cell Death Pathways in Cancer. Nat. Cancer.

[33] Jolly M., Ware K.E., Gilja S., Somarelli J.A., Levine H. (2017). EMT and MET: Necessary or Permissive for Metastasis?. Mol. Oncol..

[34] Khan S.U., Fatima K., Malik F., Kalkavan H., Wani A. (2023). Cancer Metastasis: Molecular Mechanisms and Clinical Perspectives. Pharmacol. Ther..

[35] Sorenson C.M. (2004). Bcl-2 Family Members and Disease. Biochim. Biophys. Acta (BBA) Mol. Cell Res..

[36] Bouillet P., Strasser A. (2002). BH3-Only Proteins—Evolutionarily Conserved Proapoptotic Bcl-2 Family Members Essential for Initiating Programmed Cell Death. J. Cell Sci..

[37] Moravcikova E., Krepela E., Donnenberg V.S., Donnenberg A.D., Benkova K., Rabachini T., Fernandez-Marrero Y., Bachmann D., Kaufmann T. (2017). BOK Displays Cell Death-independent Tumor Suppressor Activity in Non-small-cell Lung Carcinoma. Int. J. Cancer.

[38] Kale J., Osterlund E.J., Andrews D.W. (2018). BCL-2 Family Proteins: Changing Partners in the Dance towards Death. Cell Death Differ..

