# Supplementary material for: BCL-B Promotes Lung Cancer Invasiveness by Direct Inhibition of BOK
Source: Cells. 2025 Feb 9;14(4):246. doi: 10.3390/cells14040246 (PMC11853756; doi:10.3390/cells14040246)
Supplement: Supplementary file 1 [file cells-14-00246-s001.zip › cells-3410469-supplementary.pdf]

Supplementary Figures

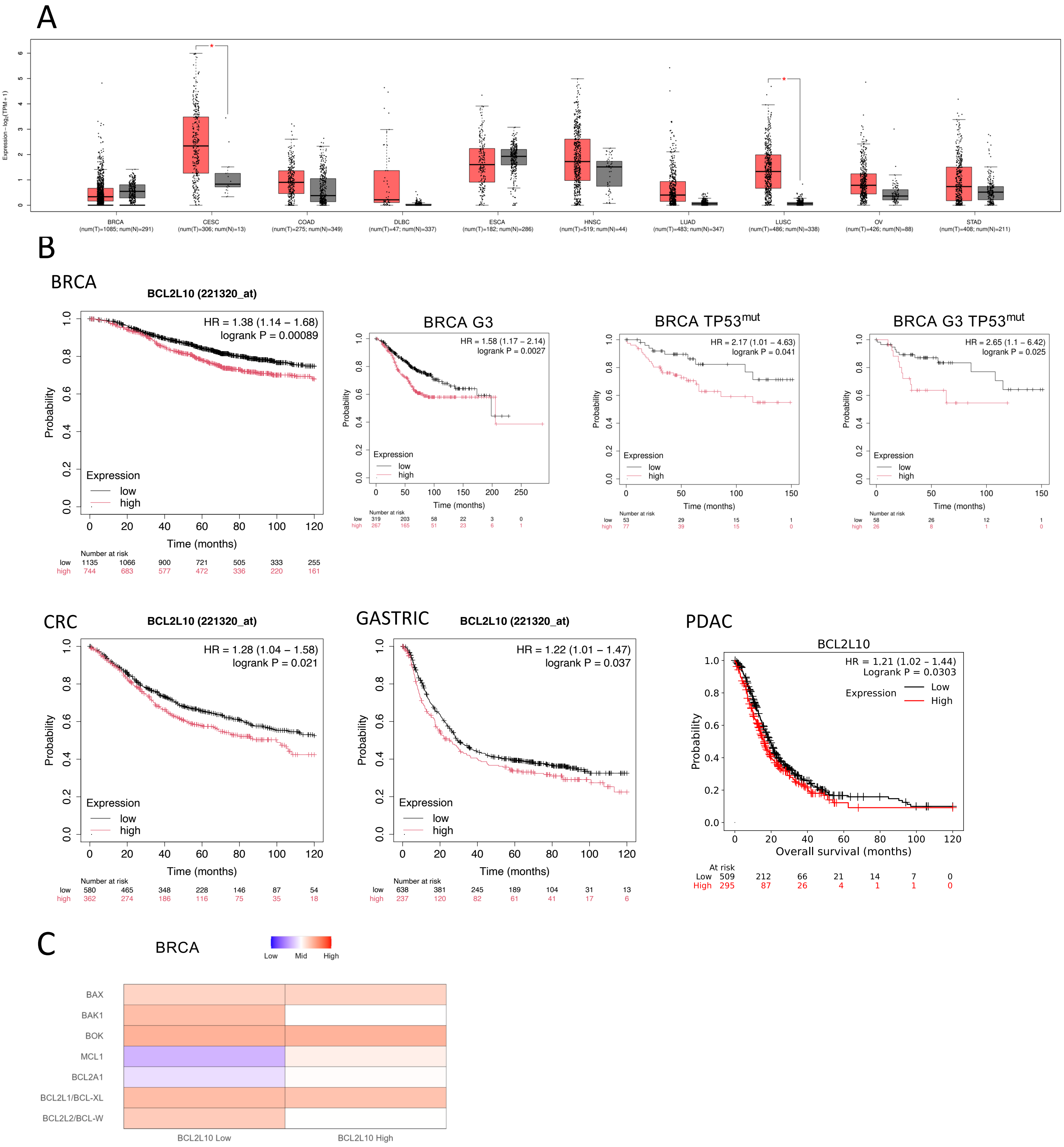

Supplementary Figure S1. BCL-B is highly expressed in cancers and correlates with survival in select tumor types

(A) BCL-B expression in various tumors: breast cancer (BRCA), cervical squamous (CESC), colon adenocarcinoma (COAD, CRC), diffuse lymphoid B-cell Lymphoma (DLBC), esophageal carcinoma (ESCA), head and neck squamous carcinoma (HNSC), lung adenocarcinoma (LUAD), lung squamous carcinoma (LUSC), ovarial cancer (OV) and stomach adenocarcinoma (STAD, gastric) (T, red bars) compared to normal tissue (N, grey bars). * p <0.05 (ANOVA). LUAD and LUSC data are same as in Figure 1A and have been added here for comparability.

(B) Kaplan Meier survival curves of patients with indicated cancers generated with Kaplan-Meier-Plotter generated with "auto select best cutoff" of BCL-B expression from various databases as listed in the references. Hazard ratio (HR) and p-values are indicated.

(C) Heatmap showing normalized RSEM (RNA-Seq by Expectation-Maximization) data from TCGA database on BCL-2 family protein expression in BRCA.

Supplementary Figures

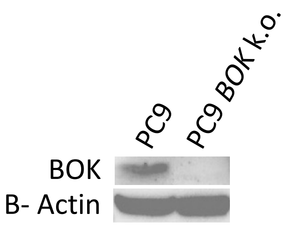

Supplementary Figure S2. Generation of *BOK* k.o. cells

Immunoblot of BOK and Actin in WT and generated BOK k.o. PC9 cells.

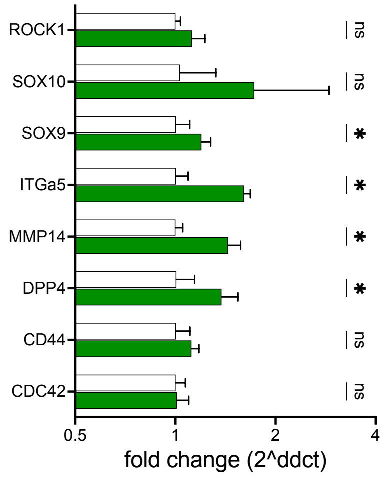

Supplementary Figure S3. DTPs express EMT markers

Expression of various EMT related transcripts in PC9 control (white) and DTPs (green) generated after 4h treatment with ABT737 1,5µM and S6 3µM. Statistical analysis was performed using unpaired student's t-test. ns, not significant ($P > 0.05$), $^*P < 0.05$.
